# Letter to the Editor in response to the article “Semaglutide and post kidney transplantation in patients with diabetes”

**DOI:** 10.1016/j.jcte.2024.100358

**Published:** 2024-07-02

**Authors:** Mai Hussein, Youssef MK Farag

**Affiliations:** a Boston University School of Public Health, Boston, MA, USA; b Alexandria Clinical Research Administration, Ministry of Health and Population, Egypt; c Department of Epidemiology, Johns Hopkins Bloomberg School of Public Health, Baltimore, MD, USA; d Postgraduate Medical Education, Harvard Medical School, Boston, MA, USA; e Alexion Pharmaceuticals, AstraZeneca Rare Disease Unit, Boston, MA, USA

To the editor,

We carefully read the article by Mahzari *et al* on the safety and efficacy of semaglutide in patients with post-kidney transplant patients with T2DM (T2DM) or Post-Transplant Diabetes Mellitus (PTDM) [1]. We applaud the authors for their innovative clinical practice in exploring the use of semaglutide in this patient population, and their proactive documentation of their experience in publishing this to the clinical and scientific community. We would like to highlight few methodologic limitations that may guide the readers to interpret the findings of this study.

First, all the patients who were included in this study were exposed to semaglutide, with no comparison. Given the descriptive nature of the study and the absence of a comparison group, it may be inappropriate to draw casual conclusions and definitive statements from this study related to the safety, tolerability, and effectiveness of semaglutide in this patient population. It would have been more appropriate if the authors included a comparison group who were not exposed to semaglutide to allow for a more robust assessment of the drug’s safety and effectiveness. Thus, any changes, or lack thereof, in the clinical or laboratory variables in this group of patients all of whom are exposed to semaglutide can very much be attributed to regression to the mean or the natural progression, or regression, of the disease. Furthermore, all these variables have natural variability and imprecision in the measurement which could all contribute to observing the results that are reported in this study.

Second, it is not clear to the readers why the authors decided to combine both adolescents and adults in one dataset and not to analyze them separately. Adolescents exhibit different biological and physiologic characteristics compared with adults as related to the etiology of kidney disease that led to kidney transplant, and their demographics are likely to be different as well. Similarly, it may not have been justified to combine patients with pre- and post-transplantation type 2 diabetes.

Third, Rybelsus (oral tablets with doses ranging 3-14mg) [2], Wegovy (subcutaneous injection for obesity management with doses ranging 0.25–2.4mg) [3], and Ozempic (subcutaneous injection for diabetes management with doses ranging 0.25-2mg) [4] are three formulations containing semaglutide. Since each of these formulations have different pharmacokinetic profile and, depending on the route of administration, the efficacy and effectiveness may be variable, and it would have been more informative if this information was available to readers.

Fourth, the initial dose level of semagultide, regardless of route of administration, is known to be non-clinically effective. The Cmax of semaglutide is reached 1–3 days post-dose across all formulations, with steady-state exposure achieved after 4–5 weeks of consistent administration. It might have been more valuable to assess any changes in the clinical and laboratory variables after 5 weeks each patient has reached the steady-state level per label.

Fifth, it is challenging to make definitive conclusions regarding the drug’s safety profile in this patient population with the small sample size. Furthermore, it would have been more valuable to provide information on how the reported side effects were measured; whether they were self-reported by patients or assessed by healthcare providers, and whether any criteria used for grading the severity of side effects. A detailed assessment of the clinical consequences of these side effects were not described e.g. changes in medication (e.g., discontinuation, dose reduction), and the resolution of symptoms over time.

Sixth, the authors conclude that their findings are consistent with conclusions drawn from previous studies regarding the safety and effectiveness of GLP-1 receptor agonists (GLP-1RAs) in controlling blood glucose and reducing weight in renal transplant recipients. We disagree. Given the aforementioned concerns, we believe that this study neither confirms nor refutes the findings of earlier studies.

Finally, the use of efficacy in this context is inappropriate, which is more appropriate in the context of a randomized controlled trial. We rather use the term “effectiveness” in observational studies. We look up to the authors to lead and encourage the scientific community to continue their innovation in leading randomized controlled trials for the use of GLP1-RAs in this patient population to provide definitive answers to sorely needed questions. This shall facilitate bringing new therapies to often-excluded patients from clinical trials.

## Declaration of competing interest

The authors declare the following financial interests/personal relationships which may be considered as potential competing interests: Youssef MK Farag reports a relationship with Alexion Pharmaceuticals, AstraZeneca Rare Disease Unit that includes: employment. If there are other authors, they declare that they have no known competing financial interests or personal relationships that could have appeared to influence the work reported in this paper.
